# Modulation of Conflict Processing by Reappraisal: An Experimental Investigation

**DOI:** 10.3390/brainsci12050564

**Published:** 2022-04-27

**Authors:** Qian Yang, Gilles Pourtois

**Affiliations:** 1 Institute of Brain and Psychological Sciences, Sichuan Normal University, Chengdu 610066, China; 2 Cognitive & Affective Psychophysiology Laboratory, Department of Experimental Clinical and Health Psychology, Ghent University, 9000 Ghent, Belgium; gilles.pourtois@ugent.be

**Keywords:** conflict effect, negative affect, emotion regulation, reappraisal, motivation

## Abstract

Negative affect facilitates conflict processing. Here we sought to assess whether symmetrically, its downregulation by means of reappraisal could lower it. To this end, 105 participants performed the confound-minimized Stroop task eliciting negative affect that was followed by a simple reward-related visual discrimination task. Conflict processing was induced with the former task. Half of them (experimental group) were instructed to use this second task to downregulate negative affect arising from the Stroop task. The other half (control group) did not receive these appraisal-related instructions. Group comparisons showed that negative affect and the conflict effect were similar for these two groups. However, when we added and modeled the subjective ratings related to emotion regulation, we found that conflict processing significantly improved for participants who reported using reappraisal spontaneously, and this gain occurred irrespective of negative affect. These results suggest that reappraisal can influence conflict processing but this change does not depend on negative affect.

## 1. Introduction

Conflict processing is usually associated with enhanced cognitive control [1]. Recently, it has been suggested that defensive motivation and/or negative affect could also influence conflict processing [2,3,4], as conflict appears to be inherently aversive [2,5]. During conflict processing, a specific negative state could be activated, which would serve as a motivational drive for implementing additional control [6], eventually leading to improved conflict resolution subsequently. Conversely, cognitive control decreases when task-unrelated positive signals are used intermittently, which can counteract the negativity arising from conflict processing [7,8,9,10,11,12,13]. Hence, conflict processing and negative affect have close ties with each other [14].

Given the aversive quality of conflict, some researchers have suggested that conflict processing could be regarded as a form of implicit emotion regulation to some degree [15,16,17]. This strategy allows to restore a state of “cognitive comfort” and maintain homeostasis in the face of aversive events, such as conflict or error [14,17]. At the neural level, there is also indirect evidence for close ties between conflict processing and negative affect, with shared effects observed in the frontoparietal network (FPN) and the dorsal anterior cingulate cortex (dACC) [18,19]. Interestingly, the involvement of a common dACC region in these two cases could reflect their differential motivational value [14,18].

In fact, the notion that emotion regulation and conflict processing share common ground is not new, but backed up by several lines of research in the existing literature. Reappraisal in particular, which is an adaptive and effective emotion regulation strategy, is closely related to a wide range of cognitive control abilities that are thought to contribute indirectly to maintaining psychological well-being [20,21]. More specifically, it has been shown that distraction by (negative) emotional stimuli on conflict processing is less pronounced for participants who tend to use reappraisal as an emotion regulation strategy [22]. Moreover, if negative stimuli are paired with conflict (during a training phase), whose resolution requires cognitive control, participants are more likely to subsequently use reappraisal spontaneously as an emotion regulation strategy during a test phase [23]. In this sense, cognitive control can bolster reappraisal.

Whereas the studies mentioned above suggest that cognitive control can influence reappraisal, other studies have focused on the reverse modulatory effect, namely, the influence of reappraisal on cognitive control during conflict processing. For example, a reduced Stroop effect has been reported as a result of using reappraisal [24]. Moreover, we recently found that reappraisal weakened conflict adaptation [25], which reflects trial-by-trial adjustments in cognitive control [26]. More specifically, we found that participants who reported using reappraisal as the preferred emotion regulation strategy in their daily life (as estimated using the emotion regulation questionnaire—ERQ [20]) had a lower conflict adaptation effect compared with those who did not. Hence, reappraisal, when conceived as a disposition, appeared to lower conflict adaptation.

Although these studies provide some preliminary evidence regarding a possible association between emotion regulation and conflict processing, negative affect manipulated in them was deemed “incidental” due to being orthogonal or somewhat separated from conflict processing [2]. In this context, we believe it is important to note that conflict processing could be regarded as a form of implicit emotion regulation as long as negative affect is actually “integral” (i.e., directly part of, or at least strongly connected with, conflict processing), as opposed to “incidental” only [2]. However, in this case (integral negative affect), it remains largely unknown whether implicit emotion regulation could alter conflict processing. The goal of the current study was to explore this important question, and more specifically, to assess if reappraisal (which is a common emotion regulation strategy [27]), when unlocked using specific instructions during the experiment, could alter cognitive control (i.e., conflict processing).

To this end, we used the confound-minimized Stroop task (the first task) combined with punishment-related negative feedback contingent on task performance to elicit negative affect (see also [28,29], for similar manipulations). Crucially, the Stroop task was followed by a simple and seemingly unrelated visual discrimination task (the second task) where participants received a majority of positive feedback (i.e., reward) contingent on task performance. Half of the participants (experimental group) were encouraged to use it proactively as a means to downregulate negative affect that could result from the Stroop task (the first task). For these participants, reappraisal was mentioned explicitly via instructions. The other half of the participants (control group) performed the same two tasks, in the same order, however, they were not instructed to use reappraisal.

We examined if reappraisal could influence cognitive control, as reflected by a different conflict effect in the experimental group compared with the control group. More specifically, we hypothesized that conflict processing could decrease in the experimental group compared with the control group. Moreover, given the specifics of this new emotion regulation procedure, which remains largely implicit compared with strategies or tactics in the existing literature where emotion regulation is more explicit and cue or event-based [27,30], we also expected some inter-individual differences to arise in the two groups concerning the willingness, use, and success of this newly devised emotion regulation strategy based on the connection (at the emotional level) between these two tasks. These inter-individual differences and their possible effect on conflict processing (i.e., the conflict effect) were therefore also examined using exploratory analyses.

## 2. Methods

### 2.1. Participants

We recruited one hundred and five participants (all native Dutch speakers). The sample size was calculated based on a priori power analysis to detect a small effect size (partial eta squared of 0.04) with 85% power using a between-subjects factorial design, which indicated a required sample size of 102. Fifty-four of them were randomly assigned to the experimental group while fifty-one were assigned to the control group. Six participants (two from the experimental group and four from the control group) were excluded from further data analyses due to low accuracy (lower than 60%), and another one from the experimental group was excluded too because he did not complete the subjective ratings. Accordingly, the final sample consisted of ninety-eight participants; fifty-one in the experimental group (mean age = 22.4 years, *SD* = 3.3, 14 males) and forty-seven in the control group (mean age = 23.1 years, *SD* = 2.2, 13 males). Participants in the experimental group were compensated with EUR 20–22, and those in the control group were compensated with EUR 9–10 for their participation (EEG was also recorded for the participants in the experimental group, which explains why they eventually received a larger compensation than those in the control group). All participants had normal or corrected-to-normal vision and reported no history of psychiatric or neurological disorders.

### 2.2. Stimuli and Task

Participants were seated in front of a computer monitor and asked to perform two different tasks in two different blocks, namely, a speeded Stroop task and a simple visual discrimination task. The Stroop task was the main one and served to measure the conflict effect. This Stroop task was challenging for participants and increased substantially negative affect because negative feedback associated with monetary loss was provided upon incorrect or slow responses at the single-trial level, which occurred in about half of the trials [28]. The visual discrimination task was used to buffer the rise in negative affect resulting from the Stroop task, especially for the experimental group (see here below). Unlike the Stroop task, it was characterized by the delivery of a large amount of positive performance feedback (i.e., reward) throughout.

In the Stroop task, the Stroop stimuli consisted of four words (in Dutch) (“rood”/red, “blauw”/blue, “groen”/green, or “geel”/yellow; font size, 30 points) presented in one out of four possible colors (red, RGB: 255, 0, 0; blue, RGB: 0, 176, 240; green, RGB: 0, 255, 0; and yellow, RGB: 255, 255, 0). However, for a given participant, each word was presented in only two of the four possible hues (see below). To rule out contingency learning, a four-alternative forced choice (4-AFC) task was used [31,32], where two pairs of S-R were created arbitrarily to balance congruent and incongruent trials. Each pair consisted of two words and two colors such that incongruent trials were created for the (incompatible) word-color association within each pair, but not across pairs. According to this rule, 8 stimuli types were created in total (instead of 16 if all combinations were constructed), corresponding to 4 stimuli for congruent trials and 4 stimuli for incongruent trials. Each word was presented equally often in the congruent and incongruent colors in each block within each mapping [33]. To rule out feature repetitions across successive trials, the stimuli were systematically alternated across successive trials to ensure that there was no stimulus (or response) repetition for both goal-relevant (color) and goal-irrelevant (meaning) dimensions. On each and every trial, participants were instructed to identify the color in which the word appeared (i.e., color naming task) as fast and accurately as possible by using four predefined keys with corresponding colors (red, blue, green, and yellow) of a response box. To do so, participants used their left middle finger to respond to the red color, left index finger to the blue color, right middle finger to the yellow color, and right index finger to the green color.

**Figure 1:**
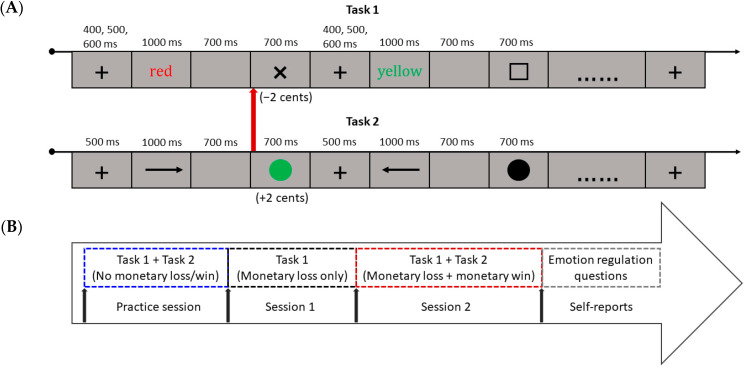
Task and experimental procedure. (**A**) For task 1 (speeded Stroop task), each trial started with a fixation cross, followed by the Stroop stimulus. A blank screen ensued, before the performance-contingent feedback was presented (being either negative or neutral). Negative feedback (a black cross) led to monetary loss, while there was no consequence for neutral feedback (a black square). For task 2 (visual discrimination task), each trial started with a fixation cross, followed by an arrow pointing either to the left or to the right. After that, a blank screen was shown, followed by the performance-contingent feedback (being either positive or neutral). Positive feedback (a green dot) led to monetary win, while there was no consequence for neutral feedback (a black dot). (**B**) General structure of the experimental procedure, consisting of four successive phases.

Each trial started with a fixation cross that was used as intertrial interval (ITI), with a mean duration of 500 ms (range: 400, 500, and 600 ms). After the fixation cross, the Stroop stimulus was presented in the middle of the screen for 1000 ms or until a response was given, followed by a blank screen shown for 700 ms, before either a negative feedback signaled by a black cross was provided if the response was incorrect or too slow (i.e., falling above the response deadline corresponding to an arbitrary time limit; see here below), or a neutral feedback signaled by a black square was provided if the response was correct and fast enough (i.e., falling below this time limit). Importantly, participants were informed beforehand that each negative feedback received would be converted to a 2-cent monetary loss, while neutral feedback would not be associated with a specific consequence (see Figure 1A). Hence, punishment motivation was selectively increased. With regard to the time limit, we used an algorithm validated previously that enforces fast responding [34] and ensures a balanced proportion of negative and neutral feedback on average without yielding excessive frustration. Unbeknownst to the participants, the reaction time (RT) cutoff was updated on a trial-by-trial basis to deal with unwanted fatigue or habituation effects throughout the experimental session.

Following the Stroop task, participants performed a simple visual discrimination task, where they were asked to carry out a two-alternative forced choice (2-AFC) task regarding the direction of an arrowhead. On each trial, participants were instructed to respond according to the direction of the arrowhead (pointing either to the left or right side) using the digits “9” and “0” of the keyboard. To do so, participants used their right index finger to press “9” and right middle finger to press “0”. Each trial started with a fixation cross that was used as the ITI and presented on the screen for 500 ms. After the fixation cross, the arrow was presented in the middle of the screen for 1000 ms or until a response was given, followed by a blank screen shown for 700 ms, before either a green or a black dot was presented (i.e., feedback), for correct and incorrect response, respectively (see Figure 1A). Participants were informed beforehand that each positive feedback received would be converted to a 2-cent monetary gain. Hence, only correct responses were rewarded, while incorrect ones were not punished. As this simple visual discrimination task was mostly used to buffer or repair negative affect resulting from the preceding Stroop task (especially in the experimental group), only accuracy but not speed was used to determine performance feedback. We aimed at getting a large amount of positive feedback associated with monetary gain for each participant.

### 2.3. Procedure

Before the start of the experiment, all participants gave informed consent and performed a practice session to become familiarized with the Stroop task (20 trials) and visual discrimination task (20 trials). During practice, punishment motivation (Stroop task) and approach motivation (visual discrimination task) were not elicited. Following practice, the experiment began and was divided into two sessions. The first session served as baseline, during which the Stroop task (*n* = 101 trials) was executed and punishment motivation introduced and elicited (unlike the practice, incorrect or slow responses were associated with monetary loss in session 1). The second session included alternations between the Stroop task and the visual discrimination task, in this specific order. More specifically, participants first performed 81 trials of the Stroop task (with punishment motivation) before they completed 40 trials of the visual discrimination task. This procedure was repeated four times (i.e., there were four combinations of the two tasks, with short self-paced breaks allowed in between). For the visual discrimination task, the stimuli were presented in random order (see Figure 1B).

For the two groups, the exact same instructions were used for both practice and session 1. However, for session 2, different instructions were given to the two groups. For the experimental group, participants were encouraged to reappraise negative affect resulting from the Stroop task while performing it, using (the prospect of) positive feedback received later with the subsequent visual discrimination task. In this group, instructions emphasized the putative association between the amount of negative feedback received with the Stroop task and the amount of positive feedback gleaned with the visual discrimination task, whereby the second one (reward) somehow canceled out the first one (punishment). In comparison, for the control group, these two tasks were introduced as separate, and not connected to one another whatsoever. Accordingly, unlike the participants of the experimental group, the participants of the control group were not encouraged to regulate negative affect (Stroop task) with the prospect of getting offsetting positive feedback (visual discrimination task). After session 2, we administered six specific questions meant to obtain a more fine-grained estimate of the emotion regulation strategy employed by the participants during the experiment. Two questions probed the willingness to downregulate negative affect encountered during the Stroop task. Two other questions were related to the actual use of the visual discrimination task to buffer negative affect generated by the Stroop task. Last, two questions probed the perceived success to do so. Hence, these three subscales actually referred to three different phases of emotion regulation, wherein “willingness” reflected participants’ motivation prior to regulation, “use” was related to actual regulation, and “success” was associated with the post-evaluation phase. All participants were asked to rate their willingness, use, and success to downregulate negative affect. In addition, we calculated bivariate correlations between these three variables (i.e., willingness, use, and success). Stimuli presentation and data recording were controlled using E-Prime (Version 2.0; Psychology Software Tools Inc., Sharpsburg, PA, USA).

### 2.4. Questionnaires

#### 2.4.1. Positive and Negative Affect Schedule

A Dutch version of the Positive and Negative Affect Schedule (PANAS; [35,36]) was used as a manipulation check for the increase in negative affect (and concurrent decrease in positive affect) with the Stroop task as soon as loss-related feedback was introduced (session 1). The scale consists of 20 words that describe different feelings and emotions (10 items for negative affect; 10 items for positive affect). The PANAS was administered 3 times in total: after the practice, after session 1, and after session 2. Each time, participants rated the 20 items on a 5-point scale ranging from 1—very slightly or not at all to 5—*extremely*. In addition, the order of these 20 items was alternated across these three measurement points to reduce the use of any predefined response strategy, or the anticipation of specific emotional words.

#### 2.4.2. Subjective Feelings (Negative Feedback vs. Positive Feedback)

Participants were asked to rate their dislike and like feelings toward negative (Stroop task) and positive (visual discrimination task) feedback, respectively, by means of a visual analog scale (VAS) ranging from 0 (not at all) to 100 (a lot) along a putative dislike or like continuum. For negative feedback, the VAS was administrated three times in total (i.e., once after each phase of the experiment), while for positive feedback, it was administrated twice only (i.e., after practice and session 2).

### 2.5. Data Analysis

We first performed standard group comparisons (i.e., experimental vs. control) for all dependent variables (i.e., conflict effect, emotion regulation score, PANAS, and subjective feelings). Second, in order to investigate whether the perceived willingness/use/success of emotion regulation could modulate conflict processing, PANAS, and subjective feelings, we added in an exploratory analysis of the emotion regulation score (i.e., willingness, use, and success) as a predictor in the (generalized) linear mixed model ((G)LMM). In this exploratory analysis, inter-individual differences in emotion regulation (as revealed by these subjective ratings), irrespective of the experimental manipulation, could be considered to assess if they might relate to cognitive control (i.e., the conflict effect) or not. A standard alpha level of 0.05 was used for all statistical tests (see Table 1 for the summary of the expected and actual (indicated in brackets) outcome for the data analysis). All data are made publicly available via the Open Science Framework (https://osf.io/gybr9/, accessed on 10 April 2022).

### 2.6. Manipulation Checks

First, using an independent-sample t test, we compared the two groups for the emotion regulation score. Second, using the linear regression model, we added the sum scores of PANAS as a dependent variable, while the group (experimental vs. control), phase (practice vs. session 1 vs. session 2) and affect (negative vs. positive) were used as independent variables (i.e., predictors). Similarly, for the subjective ratings, the mean values of dislike feelings for negative feedback and of like feelings for positive feedback were added as dependent variables, while the group (experimental vs. control) and phase (for dislike feelings: practice vs. session 1 vs. session 2; for like feelings: practice vs. session 2) were added as independent variables (i.e., predictors) in the linear regression models. In the exploratory analysis, we added the emotion regulation score as predictor in the model for the PANAS and subjective ratings, separately.

### 2.7. Conflict Processing

Behavioral data (i.e., accuracy and reaction time (RT)) preprocessing, visualization, and analysis were carried out in R [37], using the tidyverse, ggplot2, lme4, and dplyr packages [38,39,40,41]. For each subject separately, the outliers (over ±3SD from the mean) were excluded for the accuracy analysis; the error trials and outliers were excluded from further analysis for the RT data. Accuracy data, which are corresponding to a categorical dependent variable, were analyzed using a generalized linear mixed model (GLMM) with binomial distribution and a logit link function. RT data were analyzed using a linear mixed model (LMM) with which RT were log transformed. We used the mean-centered deviation coding for the two main factors (congruency and group).

We added the random intercept for each subject as the random effect in the model. For the fixed effects in the standard analysis, two main effects (congruency and group), and the two-way interaction (congruency by group) were added. In the exploratory analysis, we additionally added the subjective ratings (i.e., willingness, use, and success) as predictors in the model. In order to assess effects of each factor of interest (i.e., the main and interaction effects) on accuracy and RT data, we compared models with and without that fixed effect of interest using likelihood ratio tests. For each comparison, both models included all other fixed effects that could presumably influence the results, as well as identical random effects structures.

Last, in order to assess whether conflict processing differed between the two groups (for the standard analysis) or was modulated by the emotion regulation score (i.e., willingness, use, or success) during the baseline period (i.e., session 1), we used the GLMM and LMM approach to analyze accuracy and RT data, separately.

## 3. Results

### 3.1. Standard Analysis

#### 3.1.1. Manipulation Checks

##### Emotion Regulation Score

**Figure 2:**
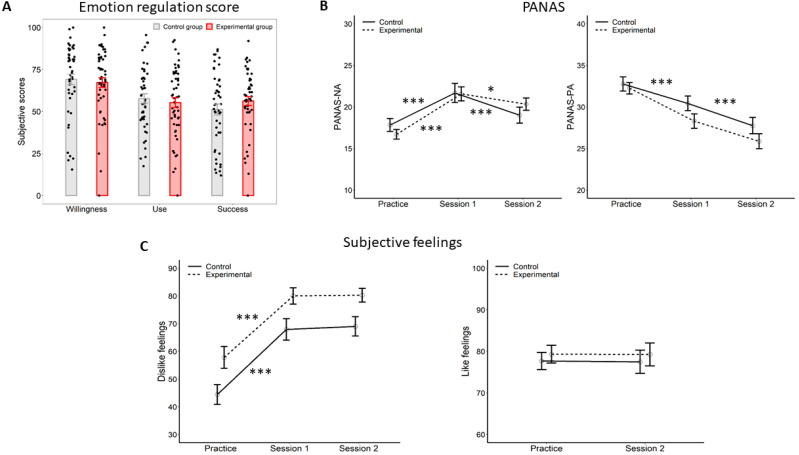
Emotion regulation scores and subjective feelings for the standard analysis (experimental group vs. control group). (**A**) Emotion regulation scores for the three subscales separately (willingness, use, or success) did not significantly differ between the two groups. (**B**) PANAS results. The two groups showed similar patterns for negative (left panel) and positive affect (right panel). (**C**) Dislike ratings of negative feedback (left panel) and like ratings of positive feedback (right panel). Vertical bars correspond to standard errors of the mean. *** *p* < 0.001, * *p* < 0.05.

The scores on the willingness, use, and success did not differ between the two groups, *ts* ≤ 1.229, *ps* ≥ 0.222, and Cohen’s *ds* ≤ 0.249 (see Figure 2A). In addition, the scores on the willingness subscale were positively correlated with those on both the use (r = 0.533, *p* < 0.001) and success (r = 0.505, *p* < 0.001) subscales. Further, the scores on the use subscale were also positively correlated with those on the success subscale (r = 0.475, *p* < 0.001).

##### PANAS

The linear regression showed that the model significantly predicted the PANAS scores, *F*(11, 576) = 44.37, *p* < 0.001, with an *R*^2^ = 0.448. More specifically, the phase significantly contributed to the model, with higher scores in session 1 than the practice and session 2, *βs* ≥ 2.680, *ts* ≥ 2.167, and *ps* ≤ 0.03. Affect significantly contributed to the model, *β* = 8.744, *t* = 7.068, and *p* < 0.001. However, the three-way interaction between affect, phase, and group did not significantly contribute to the model, *βs* ≤ 2.632, *ts* ≤ 1.085, and *ps* ≥ 0.278 (Figure 2B).

##### Dislike Feelings (Negative Feedback)

Dislike ratings were significantly accounted for by the model, *F*(5, 288) = 16.28, *p* < 0.001, with an *R*^2^ = 0.207. More specifically, the factor phase significantly contributed to the model, showing that dislike feelings in session 1 were significantly higher compared with practice, *β* = 23.489, t = 4.785, and *p* < 0.001, whereas they did not differ compared with session 2, *β* = 1.095, t = 0.223, and *p* = 0.823. Group significantly contributed to the model, with higher ratings in the experimental compared with the control group, *β* = 12.104, t = 2.516, and *p* = 0.012. However, the two-way interaction between phase and group did not significantly contribute to the model, *βs* ≤ 1.293, *ts* ≤ 0.190, and *ps* ≥ 0.849 (Figure 2C, left panel).

##### Like Feelings (Positive Feedback)

The linear regression model did not explain the like ratings, *F*(3, 192) = 0.161, *p* = 0.922, with an *R*^2^ = 0.013 (Figure 2C, right panel).

#### 3.1.2. Conflict Processing

##### Accuracy

**Figure 3:**
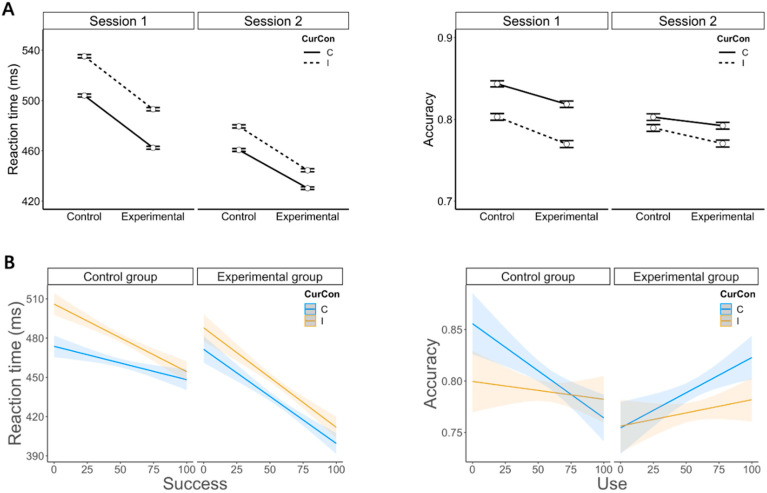
Behavioral results. (**A**) Conflict processing for the standard analysis (experimental group vs. control group). Conflict processing was similar in the two groups for RTs (left panel) and accuracy (right panel). (**B**) The influence of emotion regulation on conflict processing was modulated by the self-reported scores of emotion regulation. In the experimental group, conflict processing was not modulated by the perceived success of emotion regulation. However, in the control group, the conflict effect was reduced when the perceived success of emotion regulation increased. This effect could not be explained by a speed–accuracy tradeoff as the same trend was visible for RTs (left panel) and accuracy (right panel). Vertical bars correspond to standard errors of the mean.

The model comparison based on the fixed effects showed that the model that contained congruency was preferred over the model without this effect, indicating a significant main effect of congruency, χ^2^(1) = 14.58, *p* = 0.0001, and 95% CI = [0.053, 0.165]. The model that contained the two-way interaction between congruency and group was not preferred over the model without this effect, indicating that this two-way interaction was not significant, χ^2^(1) = 0.709, *p* = 0.399, and 95% CI = [−0.064, 0.160]. During the baseline period, conflict processing was not modulated by group either, as shown by the observation that the model with the two-way interaction between congruency and group was not preferred over the model without this effect, χ^2^(1) = 0.050, *p* = 0.822, and 95% CI = [−0.185, 0.233] (see Figure 3A, right panel).

##### RTs

The model comparison based on the fixed effects showed that the model with congruency was preferred over the model without this effect, indicating a significant main effect of congruency, χ^2^(1) = 113, *p* < 0.001, and 95% CI = [−0.017, −0.012], with faster responses for congruent than incongruent trials. The model with group was also preferred over the model without this effect, χ^2^(1) = 6.468, *p* = 0.01, and 95% CI = [−0.053, −0.007], with faster responses for the participants in the experimental than the control group. However, the model with the two-way interaction between congruency and group was not preferred over the model without this effect, χ^2^(1) = 0.154, *p* = 0.695, and 95% CI = [−0.004, 0.006]. In addition, during the baseline period, conflict processing was not modulated by group either, as shown by the observation that the model with the two-way interaction between congruency and group was not preferred over the model without this effect, χ^2^(1) = 0.821, *p* = 0.364, and 95% CI = [−0.013, 0.004] (see Figure 3A, left panel).

### 3.2. Exploratory Analysis

#### 3.2.1. Manipulation Checks

##### PANAS

When the emotion regulation score (i.e., willingness, use, and success) was added in the linear regression model, it significantly predicted the PANAS scores, *Fs* ≥ 21.78, *ps* < 0.001, with an *R*^2^*s* ≥ 0.448. However, the four-way interaction between affect, phase, group, and emotion regulation score did not significantly contribute to the model, *βs* ≤ 0.106, *ts* ≤ 0.884, and *ps* ≥ 0.376.

##### Dislike Feelings (Negative Feedback)

The emotion regulation score (i.e., willingness, use, and success) significantly predicted dislike ratings, *Fs* ≥ 7.766, and *ps* < 0.001, with an *R*^2^*s* ≥ 0.202. However, the three-way interaction between phase, group, and emotion regulation score did not significantly contribute to the model, *βs* ≤ 0.393, *ts* ≤ 1.141, and *ps* ≥ 0.254.

##### Like Feelings (Positive Feedback)

**Figure 4:**
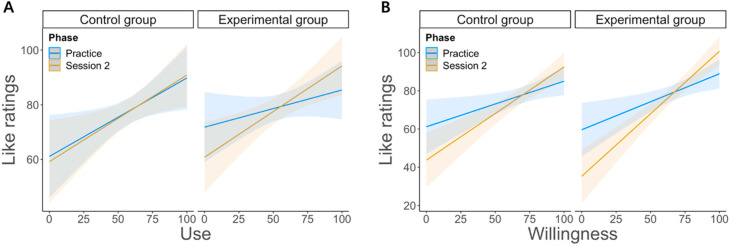
Like ratings were modulated by the perceived emotion regulation. (**A**) Like ratings increased when the perceived use increased as well. (**B**) The same was found for the perceived willingness.

The emotion regulation score (i.e., willingness, use, and success) significantly predicted like ratings, *Fs* ≥ 3.219, and *ps* ≤ 0.003, with an *R*^2^*s* ≥ 0.073. More specifically, the use (Figure 4A) and the willingness (Figure 4B) scores significantly contributed to the model, *βs* ≥ 2.287, *ts* ≥ 2.264, and *ps* ≤ 0.024; whereas the success scores did not, *β* = 0.154, *t* = 1.440, and *p* = 0.152. However, the three-way interaction between phase, group, and emotion regulation score did not significantly contribute to the model, *βs* ≤ 0.168, *ts* ≤ 0.707, and *ps* ≥ 0.480.

#### 3.2.2. Conflict Processing

##### Accuracy

The random effect showed that the variance of the subject was 0.385 (*SD* = 0.620) (see Table 2). For the fixed effects, GLMM results showed that the model with the three-way interaction between congruency, group, and use scores was preferred over the model without this effect, indicating a three-way interaction (see Table 2), χ^2^(1) = 6.874, *p* = 0.008, and 95% CI = [0.001, 0.013]. However, the model with the three-way interaction between congruency, group, and success scores was not preferred over the model without this effect, χ^2^(1) = 2.641, *p* = 0.104, and 95% CI = [−0.0009, 0.009], and the same was found for the model containing the three-way interaction between congruency, group and willingness scores, χ^2^(1) = 0.816, *p* = 0.366, and 95% CI = [−0.003, 0.008].

Since this three-way interaction (congruency by group by use score) was significant, we built two GLMMs containing two factors (congruency, use score) to assess the impact of use on congruency for the experimental and the control groups separately. For the experimental group, the model with the two-way interaction between congruency and the use scores was not preferred over the model without this effect, χ^2^(1) = 2.158, and *p* = 0.142, 95% CI = [−0.001, 0.006], indicating that the conflict effect was not modulated by the use of reappraisal in this group. However, for the control group, the model with this two-way interaction effect was preferred over the one without it, indicating that this two-way interaction between congruency and the use scores was significant, χ^2^(1) = 4.857, *p* = 0.027, and 95% CI = [−0.008, 0.0005]. As can be seen in Figure 3B (right panel), the conflict effect gradually decreased when the use scores increased. During the baseline period, none of the models with the three-way interaction between congruency, group, and willingness/use/success were significantly preferred over the ones without this effect, χ^2^(1) ≤ 0.554, and *ps* ≥ 0.456.

##### RTs

The random effect showed that the variance of the subject was 0.0032 (*SD* = 0.057) (see Table 3). For the fixed effect, LMM results showed that the model with the three-way interaction between congruency, group, and the success scores was preferred over the model without this effect, indicating that this significant three-way interaction was marginally significant (see Table 3), χ^2^(1) = 3.072, and *p* = 0.079, 95% CI = [−0.0005, 0.00002]. However, the model with the three-way interaction between congruency, group, and use scores was not preferred over the one without this effect, χ^2^(1) = 0.617, *p* = 0.432, and 95% CI = [−0.0003, 0.0002], and the same was found for the model containing the three-way interaction between congruency, group and willingness scores, χ^2^(1) = 1.384, *p* = 0.24, and 95% CI = [−0.0004, 0.0001].

To further explore the three-way interaction (congruency by group by success score), two LMMs including two factors (congruency, success score) were computed for the experimental and the control groups separately. For the experimental group, the model with the two-way interaction between congruency and the success scores was not preferred over the model without this effect, χ^2^(1) = 0.198, *p* = 0.656, and 95% CI = [−0.0003, 0.0002]. However, for the control group, the model with the two-way interaction between congruency and the success score was preferred over the model without this effect, indicating that the two-way interaction was significant, χ^2^(1) = 4.920, *p* = 0.027, and 95% CI = [0.00002, 0.0004]. As can be seen in Figure 3B (left panel), the conflict effect gradually decreased when the success scores increased. During the baseline period, none of the models with the three-way interaction between congruency, group, and willingness/use/success were significantly preferred over the ones without this effect, χ^2^(1) ≤ 1.788, and *ps* ≥ 0.181.

## 4. Discussion

The goal of the current study was to explore whether implicit emotion regulation could influence conflict processing. To test this assumption, we devised a new procedure where the participants in the experimental group could more easily establish a connection between negative affect elicited from the Stroop task and positive affect generated from the subsequent reward-related visual discrimination task, and once established, engage in indirect and implicit emotion regulation [42]. In the control condition, no such connection was installed between these two successive tasks, which could therefore be processed irrespective of each other. When using standard group comparisons, results did not show that conflict processing was different in the experimental group compared with the control group. However, given the specifics of this new procedure, some inter-individual differences in emotion regulation, and by extension conflict processing, were a priori expected to some extent. When we considered and modeled them in an exploratory analysis, we found that they significantly influenced conflict processing, and importantly, this modulation was found to be different in the experimental group compared with the control group. We hereafter discuss the implications of these new results in greater detail.

As visible from the subjective ratings, larger emotion regulation scores for the willingness, use, and success subscales were not found in the experimental group compared with the control group. This result thus suggests that the differential instructions we gave to the participants of the experimental group were not sufficient to increase at the group level the connection at the emotional level between the two tasks, which was assumed to help them use emotion regulation “implicitly”. Although reappraisal was often manipulated on a trial-by-trial basis in previous studies [27,30], here we adopted a different approach and provided participants with specific instructions at the beginning of each block, with the goal to bias implicitly conflict processing during the ensuing series of trials. Hence, this approach is probably less potent or explicit than a trial-by-trial manipulation of emotion regulation [43,44], and accordingly, reappraisal had probably a limited effect for a subset of subjects only in the experimental group.

Notwithstanding this limitation, we, however, observed that emotion regulation did influence conflict processing, yet when we considered and modeled the perceived ability of participants to use the second task to downregulate negative affect resulting from the first one (i.e., Stroop task). More specifically, we found that in the experimental group, emotion regulation had no modulatory influence on conflict processing. However, it did have a positive, albeit unspecific influence on conflict processing, as shown by faster RTs and higher accuracy for participants with larger success and use scores in this group (see Figure 3B). This suggests that the implementation of emotion regulation eased conflict processing in general, as opposed to a gain for incongruent trials selectively. In comparison, in the control group, participants who reported higher levels of successful emotion regulation showed a reduction in conflict processing (i.e., a gain for incongruent trials specifically; see Figure 3B), suggesting that they probably had higher or sharper cognitive control abilities [45]. These participants could solve conflict more efficiently than those who reported weak or no connection between the two tasks. Thus, it appears reasonable to assume that although participants assigned to the control group did not receive emotion regulation instructions, some of them could, however, “spontaneously” connect the two tasks at the emotional level, which eventually improved conflict processing. Moreover, we also found that for the two groups alike, the perceived willingness and use of emotion regulation predicted the like feelings for the reward-related feedback (as positive) during the second task (see Figure 4). This result suggests that reward processing during the second task was probably an important component for the use of implicit emotion regulation during the first task.

However, in the experimental group where a general facilitation during conflict processing was observed as a function of emotion regulation, and in the control group where this gain was restricted to incongruent trials, negative affect did not change accordingly and it was not different between these two groups (see results for PANAS as well as dislike feelings towards the negative feedback). In other words, this modulation of conflict processing, depending on emotion regulation, appeared to occur irrespective of a change or decrease in negative affect at the subjective level. This dissociation is intriguing and suggests that the reciprocal links between negative affect, emotion regulation, and cognitive control are probably not straightforward [3], and likely mediated by specific motivational or even self-regulation processes [14,22,46,47,48]. This assumption accords well with some theoretical models according to which there are strong reciprocal influences between cognitive control and self-regulation [49,50]. For example, if negative affect is not perceived or experienced as sufficiently arousing or distressing, then participants do not engage easily in self-regulation (which probably requires effort exertion; see [51]), and as a result, cognitive control remains unaffected [52]. Similarly, in our experiment, negative affect arising from the Stroop task following the encounter of frequent punishments was deemed mild or moderate only, and in this situation, it was probably not salient enough to drive a systematic change in cognitive control depending on emotion regulation, even for the participants that belonged to the experimental group [53].

Moreover, as our results for the like ratings indirectly suggest, it might be the upregulation of positive affect during the second task, rather than the downregulation of negative affect elicited by the Stroop task, which eventually drove the modulation of conflict processing by emotion regulation. In agreement with this interpretation, like feelings toward positive feedback increased with higher scores on the willingness and use subscales, which are thought to reflect a greater motivation as well as actual ability to implement emotion regulation. Hence, in our experiment, some participants probably “spontaneously” engaged in self-regulation during the Stroop task, likely because they were motivated by the prospect of reward (second task), and they had sufficient resources left to do so [54]. Moreover, if reappraisal instructions were provided to these participants (experimental group), then a general performance benefit was observed for them, whereas in the control group, where no such instructions were given, this benefit was confined to conflict resolution.

Our study only provides a first, probably imperfect attempt, based on a new experimental design, to explore more systematically the interplay between conflict processing, emotion regulation, and negative affect, and there is definitely room for improvement in the future. Moreover, some limitations warrant comment. As a matter of fact, our manipulation was not successful when assessed using standard group comparisons. Effects of emotion regulation on conflict processing were mostly found when considering inter-individual differences in the former process. In future studies, this limitation could be overcome by improving further the new emotion regulation procedure proposed in this study. For example, different (and more frequent) instructions to use emotion regulation in the experimental group could be used to increase the likelihood to observe significant group differences along this dimension, but also regarding conflict processing, which is thought to depend on negative affect and has been suggested to reflect an indirect form of emotion regulation [15,16,17]. Another caveat pertains to the emotion regulation score we have used here, and for which psychometric properties (such as validity and reliability) are currently lacking. Hence, some caution is needed in the interpretation of this emotion regulation score, and more specifically, its ability to reflect the use and success of a specific emotion regulation strategy, such as reappraisal. Future studies are needed to better delineate what this score truly captures at the emotional and regulation levels; an effort which could turn out to be beneficial to determine more precisely which specific component or process of emotion regulation is eventually susceptible to alter conflict processing. Last, we could reason that our manipulation was not successful because the speeded Stroop task we have used here was demanding or challenging for the participants, and hence there was actually little room for them to use reappraisal to downregulate negative affect arising from it. In this context, we believe that the use of refined experimental paradigms in the future could address this issue. For example, through a dedicated training procedure, the use of reappraisal could be fostered, which could create a more potent effect for its actual use subsequently during the encounter of negative affect [23].

## 5. Conclusions

To conclude, the results of this study suggest that implicit emotion regulation can influence cognitive control, and as such, they add to a growing literature in psychology and neuroscience that seeks to better connect negative affect, defensive motivation, and cognitive control with each other, and eventually explain their reciprocal interactions using a unified theoretical framework [3,18]. More than negative affect per se and/or its downregulation, our new results indirectly suggest that that motivation and ability to spontaneously engage in implicit emotion regulation may be important factors accounting for the modulation of conflict processing. Hence, we suggest that besides negative affect and emotion regulation, motivational or self-regulation processes, which are difficult to manipulate or assess and need to be better examined in future studies, probably also contribute to shape and influence cognitive control.

## Figures and Tables

**Table 1:** Summary of the expected and actual (indicated in brackets) outcome for the standard and exploratory analysis.

	Emotion Regulation Score (Willingness, Use, Success)	Dislike Feelings/ PANAS-Negative Affect	Like Feelings	Conflict Effect (Accuracy, RTs)
Standard analysis (Experimental vs. control)	Increased (no difference)	Decreased (no difference)	Increased (no difference)	Decreased (no difference)
Exploratory analysis	-	Decreased (no difference)	Increased (increased)	Decreased (decreased)

**Table 2:** Summary of random and fixed effects (ACC) when the subject-specific use scores were included in the statistical model (exploratory analysis).

**AIC**	**BIC**	**logLik**	**Deviance**	**df.resid**
30,722.3	30,797.4	−15,352.2	30,704.3	30,852
**Random effects**				
**Groups**	**Name**	**Variance**	**Std.Dev.**	
Subject	(Intercept)	0.385	0.620	
**Fixed effects**				
**Predictor**	**Estimate**	**SE**	**z-Value**	**Pr(>|z|)**
(Intercept)	1.427	0.064	22.041	<2^−16 a^
Congruency	0.115	0.028	4.034	5.47^−5 a^
Group	−0.119	0.129	−0.923	0.356 ^d^
Use-score	−0.001	0.003	−0.374	0.708 ^d^
Congruency:Group	0.043	0.057	0.760	0.447 ^d^
Congruency:Use-score	−0.001	0.001	−0.559	0.576 ^d^
Group:Use-score	0.004	0.006	0.629	0.529 ^d^
Congruency:Group:Use-score	0.007	0.002	2.625	0.008 ^b^

‘a’ < 0.001; ‘b’ < 0.05; ‘d’ > 0.1.

**Table 3:** Summary of random and fixed effects (RTs) when the subject-specific success scores were included in the statistical model (exploratory analysis).

**AIC**	**BIC**	**logLik**	**Deviance**	**df.resid**
−36,336.7	−36,258.1	18,178.4	−36,356.7	19,250
**Random effects**				
**Groups**	**Name**	**Variance**	**Std.Dev.**	
Subject	(Intercept)	0.0032	0.057	
Residual		0.0086	0.093	
**Fixed effects**				
**Predictor**	**Estimate**	**SE**	**t-Value**	**Pr(>|z|)**
(Intercept)	2.638	5.805^−3^	454.484	<2^−16 a^
Congruency	−1.397^−2^	1.359^−3^	−10.277	<2^−16 a^
Group	−2.807^−2^	1.162^−2^	−2.416	0.017 ^b^
Success-score	−4.428^−4^	2.858^−4^	−1.549	0.124 ^d^
Congruency:Group	6.257^−4^	2.718^−3^	0.230	0.818 ^d^
Congruency:Success-score	6.968^−5^	6.815^−5^	1.022	0.307 ^d^
Group:Success-score	−2.874^−4^	5.700^−4^	−0.504	0.615 ^d^
Congruency:Group:Success-score	−2.382^−4^	1.359^−4^	−1.753	0.079 ^c^

‘a’ < 0.001; ‘b’ < 0.05; ‘c’ < 0.1; ‘d’ > 0.1.

## Data Availability

All data are made publicly available via the Open Science Framework (https://osf.io/gybr9/, accessed on 10 April 2022).
